# Phosphoinositide signaling and regulation in *Trypanosoma brucei*: Specialized functions in a protozoan pathogen

**DOI:** 10.1371/journal.ppat.1008167

**Published:** 2020-01-02

**Authors:** Igor Cestari

**Affiliations:** Institute of Parasitology, McGill University, Ste-Anne-de-Bellevue, Québec, Canada; University at Buffalo School of Medicine and Biomedical Sciences, UNITED STATES

## Introduction

**Fig 1:**
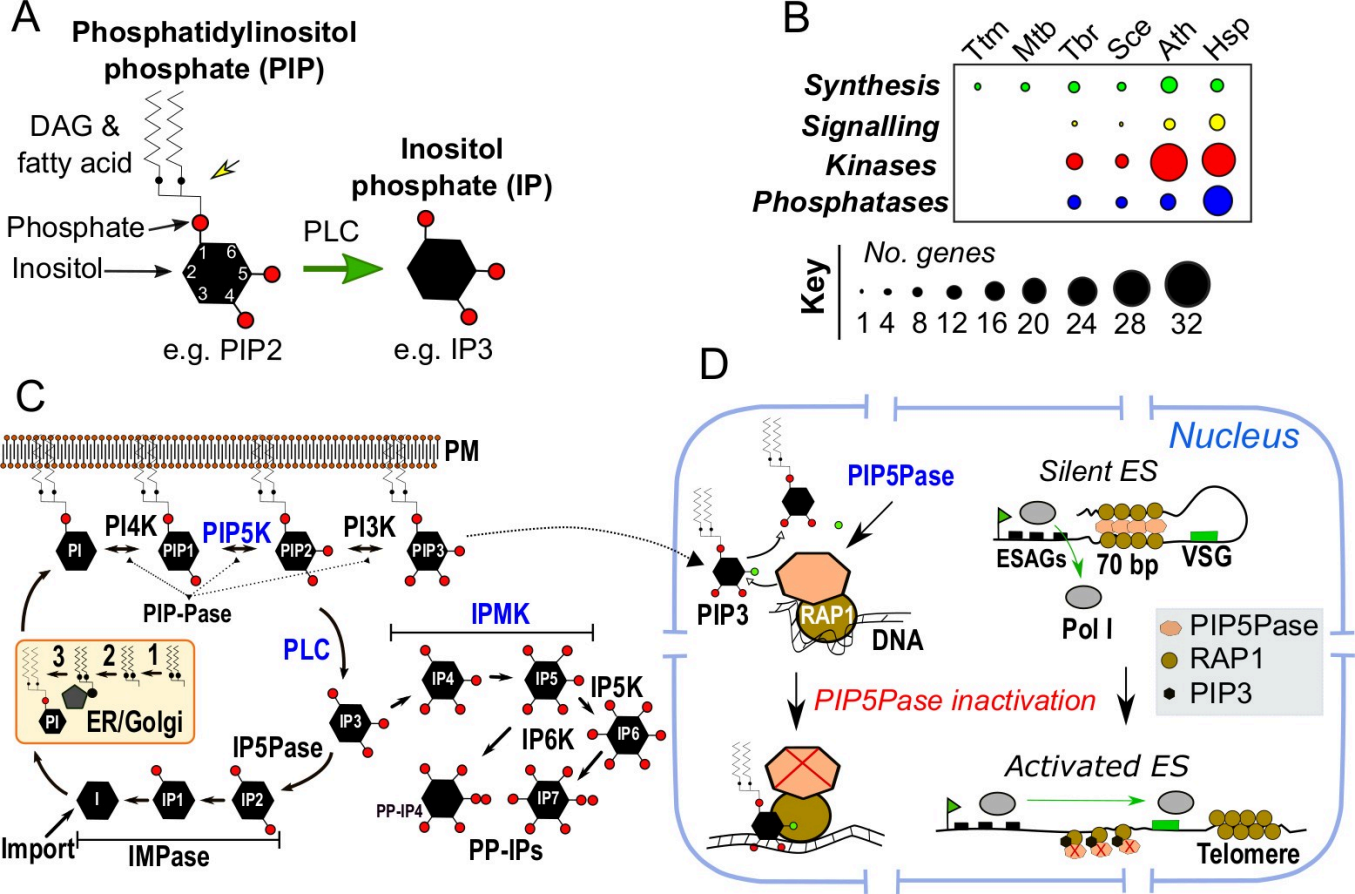
PIP and IP synthesis and regulation in *T*. *brucei*. (A) Structure of PIP2 indicated by the inositol ring (black hexagon), phosphates (red circles), and DAG with fatty acid chain. PLC cleaves PIP2 and produces diacylglycerol and IP3. Black arrows indicate phosphate and inositol. The yellow arrow indicates the site of PLC cleavage, which occurs between DAG and phosphate sn1. The green arrow indicates the directionality of the PLC reaction. (B) The number of genes involved in PIP and IP synthesis, signaling (includes PLC and IP3 receptors), and PIP and IP kinases and phosphatases in eukaryotes and prokaryotes. The size of the black circles indicates the number of genes in each category. (C) Synthesis of PIPs and IPs based on *T*. *brucei* predicted and characterized enzymes. Enzymes, whose regulatory functions are discussed here, are indicated in blue. PIP-Pase indicates enzymes that dephosphorylate PIPs at positions 3, 4, or 5 of the inositol ring. It includes PIP5Pase, whose catalytic activity is detailed below in D. Metabolite short names are used for simplicity. (D) Regulation of VSG silencing by PIP5Pase. PIP5Pase dephosphorylates the 5-phosphate (green circle) of PIP3 and prevents this metabolite binding to RAP1, which preserves RAP1 function (and likely other proteins) in ES chromatin organization. Catalytic inactivation of PIP5Pase results in PIP3 binding to RAP1, which affects ES chromatin organization and results in transcription of VSG genes. 1, diacylglycerol kinase; 2, cytidine diphosphate-diacylglycerol synthase; 3, phosphatidylinositol synthase; 70 bp, 70 base pair repeats; Ath, *Arabidopsis thaliana*; DAG, diacylglycerol; ER, endoplasmic reticulum; ES, expression site; ESAG, expression site associated genes; Hsp, *Homo sapiens*; I, myo-inositol; IMPase, inositol monophosphatase; IP, inositol phosphate; IP1, D-myo-inositol 1-monophosphate; IP2, D-myo-inositol 1,4-diphosphate; IP3, D-myo-inositol 1,4,5-triphosphate; IP4, D-myo-inositol 1,3,4,5-tetrakisphosphate; IP5, D-myo-inositol 1,2,3,4,5-pentakisphosphate; IP5Pase, inositol polyphosphate 5-phosphatase; IP6, D-myo-inositol 1,2,3,4,5,6-hexakisphosphate; IP6K, inositol hexakisphosphate kinase; IP7, D-myo-inositol 5-diphospho 1,2,3,4,6-pentakisphosphate; IPMK, inositol polyphosphate multikinase; Mtb, *Mycobacterium tuberculosis*; PIP, phosphatidylinositol phosphate; PIP1, phosphatidylinositol 4-phosphate; PIP2, phosphatidylinositol 4,5-biphosphate; PIP3, phosphatidylinositol 3,4,5-triphosphate; PIP5K, phosphatidylinositol phosphate 5-kinase; PIP5Pase, phosphatidylinositol phosphate 5-phosphatase; PIP-Pase, phosphatidylinositol phosphate phosphatases; PLC, Phospholipase C; PM, plasma membrane; Pol I, RNA polymerase I; PP-IP4, D-myo-inositol 5-diphospho 1,3,4,6-tetrakisphosphate; RAP1, repressor-activator protein 1; Sce, *Saccharomyces cerevisiae*; sn1, unimolecular nucleophilic substitution; Tbr, *T*. *brucei*; Ttm, *Thermus thermophilus*; VSG, variant surface glycoprotein.

*Trypanosoma brucei* is a single-celled protozoan pathogen that causes human and animal trypanosomiasis and incurs devastating health and economic burdens in Africa. Together with the related parasites *T*. *cruzi* and *Leishmania spp*., which cause Chagas disease and leishmaniasis, respectively, over 8 million people are affected annually worldwide [1]. These parasites alternate between a mammalian host and the insect vector and undergo extensive developmental changes during their life cycle, including changes in surface coat, gene expression, metabolism, and organelle morphology and function. They also have elaborate mechanisms of gene regulation that control the expression of genes involved in host immune evasion during infection. The control of developmental changes and immune evasion mechanisms entails a complex network of signaling and regulatory processes that includes phosphatidylinositol (PI) phosphates (PIP, also called phosphoinositides) and inositol phosphates (IP) [2–8]. PIPs and IPs are ubiquitous in eukaryotes and consist of a subset of molecules containing mono or poly phosphorylated inositol (Fig 1A). Whilst PIPs are a class of phospholipids generally associated with cellular or organellar membranes and produced via phosphorylation of PI, IPs are soluble molecules produced as a result of PIP hydrolysis by phospholipase enzymes. PIPs and IPs interact with proteins or RNA and regulate numerous cellular functions in eukaryotes. As detailed below, these metabolites and related enzymes function as a regulatory system with essential roles in *T*. *brucei* metabolism and development [6], trafficking and organelle biogenesis [9–11], Ca^2+^ signaling [12], and immune evasion mechanisms [5, 7].

## From structural molecules to regulators

The *T*. *brucei* genome encodes four enzymes involved in the synthesis of inositol and PI, one inositol symporter, 23 PIP or IP kinases and phosphatases, one phospholipase C (PLC), and one inositol trisphosphate (IP3)/ryanodine receptor (IP3RyR) [13] (Fig 1B). *T*. *brucei* synthesizes PI in the endoplasmic reticulum (ER) and Golgi [14, 15], which is then distributed to other subcellular compartments by mechanisms yet unknown. At the plasma membrane inner leaflet, PLC cleaves phosphatidylinositol 4,5-bisphosphate (PIP2) and generates diacylglycerol and IP3 (Fig 1A and 1C), and the latter is further phosphorylated or dephosphorylated by IP kinases and phosphatases, respectively [6, 16, 17] (Fig 1C). This set of synthesis, cleavage, and modifying enzymes (hereafter referred as PIP/IP-related proteins) produces at least 11 different PIP and IP metabolites (Fig 1C), some of which have been detected in *T*. *brucei* via immunofluorescence or mass spectrometry methods [7, 14, 15] or predicted to exist based on in vitro enzymatic studies [6, 13, 18]. *T*. *brucei* PIP and IP kinases and phosphatases with different specificities are distributed in distinct subcellular locations, e.g., plasma membrane, endosomes, and nucleus [5, 7, 9, 10, 12] (Table 1). The subcellular distribution of PIPs, IPs, and related proteins in *T*. *brucei* indicates that they function as a regulatory system in addition to their role in the synthesis of membrane or glycoconjugate structures. This is evidenced by the numerous cellular processes that are affected by knockdown or mutation of genes encoding PIP/IP-related proteins [6, 9, 10, 12, 18, 19] (Table 1). This regulatory system relies primarily on the activity of PIP and IP kinases and phosphatases, which control the phosphorylation and turnover of PIP and IP metabolites, and on the ability of these metabolites to interact with proteins and thus regulate protein function. Similar to *T*. *brucei*, other single-celled eukaryotes, such as *Plasmodium sp*., *Giardia sp*., and *Saccharomyces sp*. (baker’s yeast), seem to have a PIP/IP regulatory system (Fig 1B). Notably, this regulatory system seems to be absent in prokaryotes, which only have enzymes involved in the synthesis of inositol and PI, which are typically incorporated into glycoconjugates [20]. On the other hand, metazoans have a PIP/IP regulatory system with expanded and diversified gene content encoding PIP and IP kinases, phosphatases, and phospholipases, which likely reflects the diversity of cell types, developmental processes, signaling, and regulation in metazoans that are absent in unicellular eukaryotes [21]. This scenario indicates that PIPs, IPs, and related proteins function as a regulatory system that diversified with the complexity of eukaryotic organisms.

**Table 1:** Regulatory roles of PIP and IP enzymes in *T*. *brucei*.

Gene ID	Enzyme	Regulatory process	Localization	Reference
Tb927.4.1620	PIP5K	VSG gene exclusive expression and switching	Plasma membrane and endosomes	[7]
Tb927.11.6270	PIP5Pase	VSG gene exclusive expression	Nucleus	[5, 7]
Tb927.11.5970	PLC	VSG gene exclusive expression	Plasma membrane	[7]
Tb927.9.12470	IPMK	Metabolic switch from glycolysis to oxphos and development of BFs to PFs	Plasma membrane and cytosol	[6]
Tb927.8.2770	IP3RyR	Intracellular Ca^2+^ from acidocalcisomes	Acidocalcisomes	[12]
Tb927.4.1140	PI4K	Protein trafficking and Golgi maintenance and structure	Golgi complex	[11]
Tb927.8.6210	PI3K	Golgi complex segregation	Golgi complex	[10]
Tb927.11.1460	PI3P5K	Protein trafficking and multivesicular body degradation	Endosome and lysosomes	[9]

Gene IDs are from TriTrypDB. IP3RyR, inositol trisphosphate/ryanodine receptor; IPMK, inositol polyphosphate multikinase; PI3K, phosphatidylinositol 3-kinase; PI3P5K, phosphatidylinositol 3-phosphate 5-kinase; PI4K, phosphatidylinositol 4-kinase; PIP5K, phosphatidylinositol phosphate 5-kinase; PIP5Pase, phosphatidylinositol phosphate 5-phosphatase; PLC, phospholipase C

## IP signaling and regulation: Beyond second messenger function

In metazoans, IP3 functions as a second messenger, which controls Ca^2+^ release from the ER via an IP3RyR and activates downstream signaling cascades [22]. IP3RyRs have been identified in trypanosomes and paramecium [12, 23]. However, they are localized in vacuoles that store Ca^2+^ and function in osmoregulation. Interestingly, IP3RyRs have not been identified thus far in other protozoans such as *Plasmodium sp*. or *Giardia sp*. Yeast also lacks IP3RyRs, and intracellular Ca^2+^ levels are regulated via a vacuolar transient receptor potential channel that functions in osmoregulation [24]. Hence, IP3 may function in osmoregulation in some protozoans and perhaps evolved functions that are specific to the biology of these organisms.

*T*. *brucei* inositol polyphosphate multikinase (IPMK) phosphorylates IP3 and generates inositol tetra (IP4) and pentakisphosphate (IP5) [6, 13], which are further phosphorylated into inositol hexakisphosphate (IP6) and inositol pyrophosphates (PP-IPs) [18]. These IPs play essential roles in trypanosomes, as evidenced by the finding that knockdown or catalytic mutations of *T*. *brucei* IPMK affect survival, development, and metabolism (discussed below) [6, 13] (Table 1). IPMK inhibitors also affect *T*. *cruzi* amastigote proliferation [13], and knockdown of *T*. *cruzi* IP3RyR affects growth, survival, and differentiation [19]. The molecular basis underlying IP regulatory function in *T*. *brucei* is likely to function analogous to their yeast and metazoan counterparts, i.e., by interacting with proteins and thus regulating protein activity, interactions, or localization [16, 25–27]. *T*. *brucei* has several proteins that bind to IP3 or IP4 [6], most of which function in metabolism, protein synthesis and turnover, motility, and signal transduction [6]. The control of IP phosphorylation, and thus their association with proteins, provides a reversible and fast regulatory mechanism to control protein function. The characteristics of this system may be essential to regulate cellular processes in response to rapid environmental or physiological changes during parasite development and infection.

## Nuclear PIs: Transcriptional control of variant surface glycoprotein genes and antigenic variation

*T*. *brucei* expresses a homogeneous surface coat of variant surface glycoproteins (VSGs) and periodically switches its expression to escape host antibody recognition in a process known as antigenic variation. This parasite selectively expresses one out of hundreds of VSG genes, which is transcribed from one of about 20 telomeric expression sites (ESs). *T*. *brucei* changes VSG expression by transcriptional switch between ESs or by VSG gene recombination (reviewed in [4]). The control of VSG-exclusive expression and switching entails a regulatory system that includes nuclear proteins, e.g., chromatin regulatory proteins, nuclear lamina proteins, and nonnuclear proteins [4]. Phosphatidylinositol phosphate 5-kinase (PIP5K) and PLC, both of which localize in the plasma membrane inner leaflet and endosomal compartments, regulate VSG allelic exclusion and switching [7]. Knockdown of PIP5K results in simultaneous transcription of all telomeric ES VSG genes. Reexpression of PIP5K resumes VSG-exclusive expression but results in switching of the VSG gene expressed by either transcriptional or recombination mechanisms. Moreover, overexpression of PLC, but not a mutant catalytic inactive version of PLC, results in transcription of silent VSG genes [7]. The involvement of these proteins in VSG silencing and switching is suggestive of a signal transduction system that is reactive to cellular changes, perhaps via external stimuli or inherent to developmental processes.

How such a system regulates silencing and switching of VSG genes is yet unclear, but it might involve the control of PIPs subcellular fluxes and levels. *T*. *brucei* expresses a nuclear phosphatidylinositol phosphate 5-phosphatase (PIP5Pase) enzyme that also controls silencing of telomeric and subtelomeric VSG genes [5]. PIP5Pase associates with repressor-activator protein 1 (RAP1) within a 0.9 megadalton protein complex, which also includes protein kinases, phosphatases, chromatin regulatory proteins, and nuclear pore proteins [5, 7]. PIP5Pase regulation of VSG silencing revolves around the control of phosphatidylinositol 3,4,5-triphosphate (PIP3) levels. PIP5Pase dephosphorylates PIP3 and prevents this metabolite from interacting with RAP1. Catalytic mutations of PIP5Pase that inhibit PIP3 dephosphorylation but do not disrupt PIP5Pase protein complex integrity result in transcription of silent VSG genes, indicating that PIP5Pase activity is essential for VSG silencing [5]. In the current model, PIP5Pase dephosphorylation of PIP3 prevents the binding of this metabolite to RAP1, which preserves the association of RAP1 (and likely other proteins within the complex) with ES chromatin and represses transcription of VSG genes (Fig 1D). Conversely, the inactivation of PIP5Pase results in PIP3 binding by RAP1, which affects RAP1 association with ES chromatin and thus chromatin organization and results in VSG gene transcription [5]. Hence, PIPs play a key role in the mechanisms that control VSG-exclusive expression and switching and provide a hint as to how VSG regulation might be integrated with regulatory and signal transduction processes.

## IP regulation of energy metabolism and development

*T*. *brucei* life stage development entails dramatic changes in energy metabolism. Mammalian infective bloodstream forms (BFs) switch energy metabolism from glycolysis, which occurs in glycosomes (peroxisomes-like organelles), to a more complex metabolism that includes mitochondrial oxidative phosphorylation in the insect stage procyclic forms (PFs) [28]. This metabolic regulation involves IPs and is coregulated with parasite development [6]. The knockdown or catalytic mutation of IPMK in BFs results in the activation of oxidative phosphorylation, which is accompanied by a 20-fold increase in parasite development from BFs to PFs [6]. After IPMK knockdown, BFs up-regulate the expression of genes encoding mitochondrial respiratory complexes, some of which are only expressed in PFs, thus producing a functional respiratory chain that generates ATP. The molecular control of *T*. *brucei* metabolic switch likely involves IPMK substrates and products, e.g., IP3 and IP4, which interact with proteins involved in metabolism, protein synthesis and degradation, and signal transduction [6]. IPMK inactivation also affects the expression of RNA-binding proteins (RBPs), some of which control the expression of stage-specific genes and are involved in parasite life stage development [6, 29, 30]. Hence, IPMKs play a key role in the regulation of *T*. *brucei* metabolic switch and development.

The IPMK role in energy metabolism is conserved among *T*. *brucei*, yeast, and mammalian cells. IPMK controls the metabolic switch from oxidative phosphorylation to glycolysis in yeast and to aerobic glycolysis in cancer cells [17], known as the Warburg effect. In human cells, IPMK regulates cell metabolism independent of its catalytic activity. IPMK interacts with 5' adenosine monophosphate-activated protein kinase (AMPK) and mammalian target of rapamycin (mTOR) and regulates glucose and amino acid signaling [31, 32]. AMPK and target of rapamycin 4 (TOR4) complexes are also involved in *T*. *brucei* metabolism and development [2, 3], but it is unknown if they associate with *T*. *brucei* IPMK. Moreover, the control of energy metabolism in *T*. *brucei* depends on IPMK catalytic activity and thus on IPs [6]. In yeast, IPs control the activity of transcription factors such as glycolytic genes transcriptional activator (GCR1) and ArgR-Mcm1 transcription complex, which are involved in the expression of glycolysis and amino acid metabolism genes [17, 25]. However, unlike in yeast, *T*. *brucei* controls gene expression posttranscriptionally by regulation of RNA stability or translation, in which RBPs play a critical role. Hence, IPMK roles in metabolic regulation likely originated early in eukaryotes, but its regulatory mechanisms diversified among eukaryote organisms. In *T*. *brucei*, the switch in energy metabolism entails a regulatory system that involves IPs and posttranscriptional control of gene expression [6].

## Conclusion and perspectives

PIPs and IPs have regulatory functions in *T*. *brucei* that are conserved with other eukaryotes [9, 10]. However, PIPs and IPs have also diversified in function to control specialized processes such as antigenic variation in *T*. *brucei* [5, 7]. Regulation of this process might be conserved in other protozoan pathogens such as *Plasmodium* and *Giardia*, which also employ antigenic variation. The finding that PIP5K and PLC control VSG expression and switching raises the question of whether a signal transduction system is involved in the control of antigenic variation. VSG switching is thought to occur stochastically and perhaps initiated by events that lead to DNA break and repair [33]. An alternative hypothesis is that initiation of VSG switching occurs by activation of a signal transduction system that involves PIPs. This process may happen in addition to PIPs function in the control of VSG-exclusive expression via regulation of protein association with telomeric ES DNA [5, 7]. In contrast to PIP roles in VSG expression, the regulation of energy metabolism by IPMK while sharing conserved features among eukaryotes has diversified in the mechanisms of regulation, with *T*. *brucei* relying heavily on posttranscriptional control of gene expression [6, 17]. These are a few examples of many processes that are likely regulated by PIPs and IPs in *T*. *brucei* and in related pathogens.

There are many fundamental questions related to PIP and IP functions that remain unknown. One such question is whether PIPs and IPs are transported from cytoplasmic organelles to the nucleus or are synthesized in the nucleus. The identification of PIP5Pase in the nucleus of *T*. *brucei* is an indication that the interconversion of these metabolites occurs in the nucleus [5, 7], and PIP and IP interconversions have also been detected in the nucleus of yeast and mammalian cells [25, 34, 35]. Nevertheless, the molecular machinery that are involved in PIPs and IPs nuclear synthesis or their transport is unknown. Furthermore, the crosstalk between PIPs and IPs with other cell signaling and regulatory systems to control specific processes, e.g., energy generation or cell development, is also poorly understood. Answer to these questions may require the identification of signaling receptors, effector proteins, and proteins that link PIPs and IPs with distinct signaling pathways, e.g., cyclic adenosine monophosphate (cAMP) signaling, and the input and output of these pathways. Although many proteins have been shown to bind PIPs and IPs [6, 27], there are only a few protein domains that are known to interact with these metabolites [36]. In addition, interactions of PIPs with RNAs have been sparingly identified [37]. It is unknown how widespread PIP and RNA interactions are in eukaryotes, but trypanosomes, which rely on posttranscriptional control processes to regulate gene expression, may be an excellent model to study the potential functions of PIPs and RNA interactions. Understanding the role of PIP and IP signaling and regulation in *T*. *brucei* may help us to gain insights on the function, regulation, and evolution of signaling pathways in pathogens and in more complex multicellular eukaryotes.
